# Editorial: Highlights of the 16th European Conference on Fungal Genetics (Innsbruck, 5-8th March 2023)

**DOI:** 10.3389/ffunb.2025.1632910

**Published:** 2025-06-13

**Authors:** Toni Gabaldón, Gustavo H. Goldman

**Affiliations:** ^1^ Life Sciences Department, Barcelona Supercomputing Center (BSC), Barcelona, Spain; ^2^ Mechanisms of Disease Program, Institute for Research in Biomedicine (IRB), The Barcelona Institute of Science and Technology, Barcelona, Spain; ^3^ ICREA, Barcelona, Spain; ^4^ Centro de Investigación Biomédica En Red de Enfermedades Infecciosas, Barcelona, Spain; ^5^ Faculdade de Ciências Farmacêuticas de Ribeirão Preto, Universidade de São Paulo, São Paulo, Brazil; ^6^ National Institute of Science and Technology in Human Pathogenic Fungi, São Paulo, Brazil

**Keywords:** fungal meeting, fungi, genetics, molecular biology, research topic

The 16th European Conference on Fungal Genetics (ECFG16) was held in the lush city of Innsbruck, the capital of Tirol, in Austria, from March 5 to 8, 2023. More than 700 people attended this conference. Given the interesting science that was showcased in the conference, we decided to organize a Research Topic highlighting some of these presentations. This brief editorial, aims to serve as a starter to this special Research Topic, highlighting the main observations of each of the contributed articles.

*Fusarium solani* is a complex and adaptable group of fungi, some of which cause disease in plants and animals. Navasca et al. investigated a curious case: a strain from sugarbeet (SB1) forming dark, gall-like structures. Using advanced Oxford Nanopore and Hi-C sequencing, the authors mapped its genome at the chromosome level. Surprisingly, SB1 has a larger genome (59.38 Mb) than typical *F. solani* strains, containing 15 chromosomes—three of which appear to be accessory and rich in repetitive sequences and duplications. These features were not seen in the closely related reference strain *F. vanettenii* 77-13-4. Duplication patterns identified by the authors suggest these extra chromosomes may boost SB1’s gene repertoire, particularly in accessory genes. A broader comparison across 12 F*. solani* genomes shows a largely saturated pan-genome and reveals no strong link between genetic makeup and lifestyle. However, genes linked to metabolic versatility—like those encoding hydrolases and oxidoreductases—are enriched in the accessory genome, pointing to a role in adaptation and diversity.

Fungal cell walls rely on chitin—a tough, flexible sugar polymer—to maintain structure and adapt to growth. Producing chitin requires enzymes called chitin synthases (CHS), which must reach the cell surface to do their job. In yeast, the protein Chs7 helps guide CHS-3 from the endoplasmic reticulum (ER) to the plasma membrane. But in filamentous fungi like *Neurospora crassa*, the story is more complex. Gonzalez-Tellez and Riquelme showed two related proteins—CSE-7 and CSE-8—act as CHS transport helpers. They found that CSE-8 is essential for moving CHS-3 out of the ER; without it, CHS-3 fails to reach growth zones like the Spitzenkörper. Deleting *cse-8* also disrupts sexual development and causes abnormal perithecia. When both *cse-7* and *cse-8* are deleted, chitin content and growth drop significantly. Under ER stress, both CSE-8 and CHS-3 mislocalize, further confirming CSE-8’s ER role. These findings suggest filamentous fungi use unique, possibly CSE-independent, routes to manage chitin synthase delivery.

Fungi are everywhere, quietly but restlessly breaking down organic matter and recycling nutrients that are essential for life. Their remarkable abilities have been harnessed in agriculture and industry—as sources of enzymes, pigments, and natural pesticides, and in food production and environmental cleanup. Yet, fungi also pose serious threats by causing infections in plants, animals, and humans. These diseases are increasingly common and harder to treat due to rising drug resistance and the scarcity of new antifungal medicines. Developing effective treatments is tricky because current antifungals often have side effects, few unique targets, and limited species specificity. Excitingly, antimicrobial proteins and peptides (AMPs), especially those produced by filamentous fungi of the Eurotiales order, show promise as next-generation antifungal agents. Holzknecht and Marx explores fungi’s dual role, the challenges in combating fungal infections, and the potential of Eurotiales-derived antifungal peptides—highlighting their structure, how they work, and the hurdles to turning them into practical treatments for agriculture and medicine.

Fungal polyketides are prized natural compounds with a wide range of medicinal uses, but producing them in filamentous fungi often yields low amounts and impure extracts. To overcome this, Bejenari et al. turned to *Yarrowia lipolytica*, a robust yeast known for its lipid-building capacity thanks to abundant acetyl- and malonyl-CoA precursors—the very starting blocks for polyketides. While previous efforts focused on simple plant enzymes, the authors explored *Y. lipolytica*’s ability to produce complex fungal polyketides by introducing genes from *Fusarium solani* and *Aspergillus hancockii* using CRISPR-Cas9. Despite attempts to boost production by enhancing lipid breakdown pathways, yields remained unchanged. Still, the yeast reached promising levels of 6-methylsalicylic acid (403 mg/L) and bostrycoidin (35 mg/L), far exceeding earlier results in baker’s yeast. This study highlights *Y. lipolytica*’s potential as a versatile platform for manufacturing valuable fungal polyketides, paving the way for improved bioproduction strategies.

Altogether, the articles compiled in this special Research Topic represent the timely and exciting science presented at ECFG16. We hope you enjoy reading it!

